# Rac2 is involved in bleomycin-induced lung inflammation leading to pulmonary fibrosis

**DOI:** 10.1186/1465-9921-15-71

**Published:** 2014-06-27

**Authors:** Narcy Arizmendi, Lakshmi Puttagunta, Kerri L Chung, Courtney Davidson, Juliana Rey-Parra, Danny V Chao, Bernard Thebaud, Paige Lacy, Harissios Vliagoftis

**Affiliations:** 1 Pulmonary Research Group and Department of Medicine, University of Alberta, Edmonton, Alberta, Canada; 2 Department of Laboratory Medicine and Pathology, University of Alberta, Edmonton, Alberta, Canada; 3 Department of Pediatrics and Women and Children’s Health Research Institute, University of Alberta, Edmonton, Alberta, Canada

**Keywords:** Lung injury, Lung fibrosis, Bleomycin, Neutrophils, Rac2

## Abstract

**Background:**

Pulmonary fibrotic diseases induce significant morbidity and mortality, for which there are limited therapeutic options available. Rac2, a *ras*-related guanosine triphosphatase expressed mainly in hematopoietic cells, is a crucial molecule regulating a diversity of mast cell, macrophage, and neutrophil functions. All these cell types have been implicated in the development of pulmonary fibrosis in a variety of animal models. For the studies described here we hypothesized that Rac2 deficiency protects mice from bleomycin-induced pulmonary fibrosis.

**Methods:**

To determine the role of Rac2 in pulmonary fibrosis we used a bleomycin-induced mouse model. Anesthetized C57BL/6 wild type and *rac2*^*-/-*^ mice were instilled intratracheally with bleomycin sulphate (1.25 U/Kg) or saline as control. Bronchoalveolar lavage (BAL) samples were collected at days 3 and 7 of treatment and analyzed for matrix metalloproteinases (MMPs). On day 21 after bleomycin treatment, we measured airway resistance and elastance in tracheotomized animals. Lung sections were stained for histological analysis, while homogenates were analyzed for hydroxyproline and total collagen content.

**Results:**

BLM-treated *rac2*^*-/-*^ mice had reduced MMP-9 levels in the BAL on day 3 and reduced neutrophilia and TNF and CCL3/MIP-1α levels in the BAL on day 7 compared to BLM-treated WT mice. We also showed that *rac2*^*-/-*^ mice had significantly lower mortality (30%) than WT mice (70%) at day 21 of bleomycin treatment. Lung function was diminished in bleomycin-treated WT mice, while it was unaffected in bleomycin-treated *rac2*^*-/-*^ mice. Histological analysis of inflammation and fibrosis as well as collagen and hydroxyproline content in the lungs did not show significant differences between BLM-treated *rac2*^*-/-*^ and WT and mice that survived to day 21.

**Conclusion:**

Rac2 plays an important role in bleomycin-induced lung injury. It is an important signaling molecule leading to BLM-induced mortality and it also mediates the physiological changes seen in the airways after BLM-induced injury.

## Background

Fibrosis as a result of lung injury arising from various known or unknown etiologies, carries a high rate of morbidity and mortality [1]. Idiopathic pulmonary fibrosis is usually diagnosed at a late stage of disease, and identification of the pathogenic steps leading to fibrosis has met with limited success [2]. Various animal models of fibrotic diseases have shown a role for inflammatory cells, including macrophages and neutrophils, as well as their secreted proteases [3-5] and superoxide-derived free radicals [6,7].

One way of understanding the cellular and inflammatory mechanisms associated with lung fibrosis has been the intratracheal application of bleomycin (BLM), an antineoplastic antibiotic, to induce a model of pulmonary fibrosis in mice [8]. BLM-induced lung injury is characterized by early recruitment of neutrophils followed by extensive fibrosis that is self-limiting. Many other cells, including mast cells and macrophages, have also been implicated in establishing inflammation in this model.

Rac2 is a member of the Rho subfamily of *ras*-related guanosine triphosphatase expressed primarily in hematopoietic cells, and is a crucial molecule regulating a variety of neutrophil and macrophage functions [9-11]. Rac2 switches from a resting, inactive GDP-bound state to an active GTP-bound state in response to receptor stimulation. The GTP-bound form of Rac2 is essential for activation of the superoxide-generating NADPH oxidase complex in neutrophils [10] and is required for exocytosis of primary azurophilic granules through polymerization of F-actin filaments required for propelling granules to the cell membrane [9,12]. Macrophages utilize Rac2, in addition to the ubiquitously expressed homolog Rac1, for maximal phagocytosis and oxidant production [11]. Disruption of the gene encoding Rac2 led to attenuated lung inflammation and injury in a model of immune complex-mediated acute lung injury [13]. Diminished lung injury in *rac2*^*-/-*^ mice with acute lung injury was associated with decreased neutrophil recruitment to the airways, reduced matrix metalloproteinase (MMP)-2 and -9 levels in bronchoalveolar lavage samples, and decreased MMP-2 and -9 immunoreactivity in airway and mucosal macrophages [13]. A small molecule inhibitor of Rac1 and Rac2 also alleviates LPS-induced acute lung injury [14].

Since neutrophils and macrophages have been shown to play an important role in the development of BLM-induced fibrosis, we hypothesized that Rac2 deficiency will protect mice from lung injury and fibrosis in response to BLM administration.

## Materials and methods

### Animals

Rac2-deficient mice (*rac2*^*-/-*^) were established by gene disruption [10] and were backcrossed for >11 generations on the C57BL/6 strain background. Rac2-deficient mice were bred in our animal facility and housed under specific virus antigen-free conditions on a 12:12-h light–dark cycle and fed autoclaved food and water as needed. Wild type (WT) C57BL/6 mice were purchased from Charles River Laboratories and were housed and maintained in the same manner as *rac2*^*-/-*^ mice. For our experiments, we used female *rac2*^*-/-*^ or C57BL/6 WT mice at 10–14 wk of age. Animal experiments were approved by the University of Alberta Health Sciences Laboratory Animal Ethics Committee (Edmonton, AB, Canada).

### BLM-induced pulmonary fibrosis

After general anesthesia with 4% isoflurane inhalation, the trachea was exposed and a single intratracheal injection of BLM sulphate (0.125 U/100 g body weight dissolved in normal saline, Sigma-Aldrich Co., MO, USA) or saline as control was administrated to C57BL/6 WT and *rac2*^*-/-*^ mice. Following the injection, mice were sutured, allowed to recover and daily monitored for morbidity and mortality.

### Lung function measurements

On day 21 after BLM intratracheal instillation, mice were weighed and lung function was measured. Each mouse was anesthetized with 0.1 ml/10 g body weight of a mixture containing xylazine (1.6 mg/ml; Bayer) and ketamine (8 mg/ml; Parnell). Following anesthesia, a tracheotomy was performed, and a polyethylene cannula was inserted. Pancuronium amide (2 mg/kg) was administered intravenously before animals were attached to a ventilator. Mice were mechanically ventilated on a computed controlled piston ventilator FlexiVent® Systems (SCIREQ, Montreal, QC, Canada) with a tidal volume of 5.5 ml/kg at a rate of 150 breaths/min.

### Lung histology, αSMA and TGFβ analysis

On days 7 or 21 after BLM administration, left lungs were cannulated, inflated with 10% neutral buffered formalin (Sigma-Aldrich, St. Louis, MO) for 5 min under a constant pressure of 20 cm of H_2_O, removed from animals, and placed in fresh 10% neutral buffered formalin for 24 h. In the case of the 21 day group, lung resection was done after lung function testing. Tissues were then embedded in paraffin blocks, and sections were cut (4 μm) and stained with hematoxylin and eosin (H&E) and periodic acid-Schiff-diastase stain (PAS-D). Staining of lung sections with Masson’s trichrome, or with antibodies for α-smooth muscle actin (αSMA) and TGFβ were performed to obtain histological evidence of fibrotic activity. Lung fibrosis was scored according to Ashcroft’s criteria as previously published [15].

### Bronchoalveolar lavage (BAL) and evaluation of cellular infiltration, neutrophil myeloperoxidase (MPO) activity, MMP and cytokine/chemokine levels

Mice were euthanized on days 3 and 7 after saline or BLM intratracheal instillation. Following blood collection by cardiac puncture, the trachea was exposed and intubated with a polyethylene catheter. Lungs were lavaged twice with 1 ml of isotonic phosphate-buffered saline (pH 7.4) and the bronchoalveolar lavage (BAL) fluid was collected. The BAL fluid was centrifuged at 300 *g* for 5 min. Total cells in the resulting pellet were counted, and cytospins of 5000 cells/sample were prepared and stained with Diff-Quick (Fisher Scientific Co, Kalamazoo, MI). Airway inflammation was assessed by counting the number of inflammatory cells in the BAL fluid as previously described [16]. BAL supernatants were kept frozen in -80°C for MMP and MPO evaluation. MMPs were evaluated by gelatin zymography as previously described [17]. MPO activity was assessed using tetramethylbenzidine (TMB) as the substrate, as previously described [9]. Cytokine and chemokine levels were also evaluated in BAL fluid at day 7 after BLM instillation.

### Biochemical analysis of lung tissue

After lung function measurements, right lungs from each animal were removed, weighed and immediately frozen on liquid nitrogen and stored at -80°C for biochemical analysis as described below.

Hydroxyproline determination was done as described [18]. In brief, lung tissue was thawed and homogenized in 1 ml of water. To precipitate protein, 125 μl of 50% trichloroacetic acid (Sigma-Aldrich) was added to the homogenate and samples were incubated on ice for 20 min. Samples were centrifuged at 300 *g* for 5 min at 4°C. Supernatants were discarded and 1 ml 12 N HCl was added to the pellet. The pellet was baked at 110°C for 24 h. The dried pellet was reconstituted with 2 ml of distilled water and 500 μl of chloramine T solution (1.4% chloramine T in 0.5 M sodium acetate and 10% isopropanol, Sigma-Aldrich) and incubated for 20 min at 24°C. Ehrlich’s/pDMAB (1 M p-diamethylaminobenzaldehyde in 70% isopropanol and 30% perchloric acid) was also added and incubated at 65°C for 15 min. A sample of 100 μl of the final reaction solution was transferred to a 96-well plate, and the absorbance for each sample was read at 550 nm in a Power Wave XS (BioTek Instruments Inc., Winooski, VT) ELISA reader. The concentration of lung hydroxyproline was calculated from a hydroxyproline standard curve and expressed as μg/gram of lung tissue.

The total collagen content of lungs was determined using homogenized right lungs and a Sircol™ Soluble Collagen Assay kit (Biocolor, Carrickfergus, UK) according to manufacter’s instructions. Data are expressed as the collagen content of the entire right lung.

### Statistical analysis

Values are expressed as mean ± SEM. Statistical differences in the mean values among treatment groups were determined by using a one-way ANOVA test with post-hoc analysis using Tukey’s multiple comparison test. In all cases, a value for *p* < 0.05 was considered statistically significant.

## Results

### Rac2 mediates airway neutrophil infiltration and MMP-9 release in the airways following BLM-induced injury

Rac2 is expressed primarily in hematopoietic cells, and has an important role in cell motility, superoxide release and degranulation in neutrophils and macrophages [9,10,19]. Rac2 also mediates acute lung injury due to immune complexes in murine models [13]. The aim of this study was to understand the role of Rac2 in BLM-induced lung injury, a model with a more prolonged time course than immune complex-mediated injury. BLM induces reversible fibrosis over 2–3 weeks; early accumulation of neutrophils [20] and macrophages [21] has been shown to be important for the transition from inflammation to fibrosis in this model.

To understand the role of Rac2 in BLM-induced lung injury, we first examined the accumulation of inflammatory cells and inflammatory mediators in the airways of mice 3 and 7 days after induction of bleomycin-induced lung injury. To do that we performed BAL on days 3 and 7 after BLM administration, and evaluated the numbers of neutrophils and macrophages, and the presence of MPO, MMP-2 and MMP-9 activity, as well as cytokine and chemokine levels.

**Figure 1:**
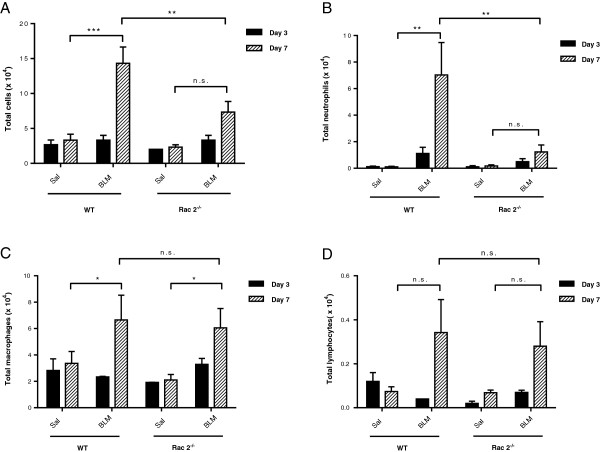
**Rac2 mediates BLM-induced airway neutrophil infiltration*. *(A)** Total cell counts, **(B)** neutrophils, **(C)** macrophages and **(D)** lymphocytes in BAL samples at 3 and 7 days after intratracheal saline or bleomycin administration to WT and *rac2*^*-/-*^ mice (n.s. not significant, *p < 0.05, **p < 0.01, ***p < 0.001, n = 5).

BAL performed on day 3 showed no significant differences in total cells, neutrophils, macrophages or lymphocytes between BLM- and saline-treated mice in WT and *rac2*^*-/-*^ groups (Figure 1A-D, n = 5). WT mice receiving BLM showed accumulation of inflammatory cells in the BAL on day 7 compared to saline-treated mice (Figure 1A). In contrast, BLM-treated *rac2*^*-/-*^ mice showed no significant accumulation of cells in BAL on day 7 (Figure 1A). While BLM induced the recruitment of neutrophils to the airways of WT mice o day 7, there were no increased numbers of neutrophils in BLM-treated *rac2*^*-/-*^ mice compared to saline treated mice on day 7 (Figure 1B). For both total cells and neutrophils there was a statistically significant difference between BLM-treated WT and *rac2*^*-/-*^ mice. The numbers of macrophages increased in BLM-treated WT and *rac2*^*-/-*^ mice on day 7 (Figure 1C) but the number of lymphocytes showed only a trend towards increase by BLM in both backgrounds (Figure 1D). There was no difference between BLM-treated WT and *rac2*^*-/-*^ mice in the number of macrophages and lymphocytes in the BAL, indicating that lack of Rac2 affects primarily neutrophil accumulation in the airways of BLM-treated mice.

**Figure 2:**
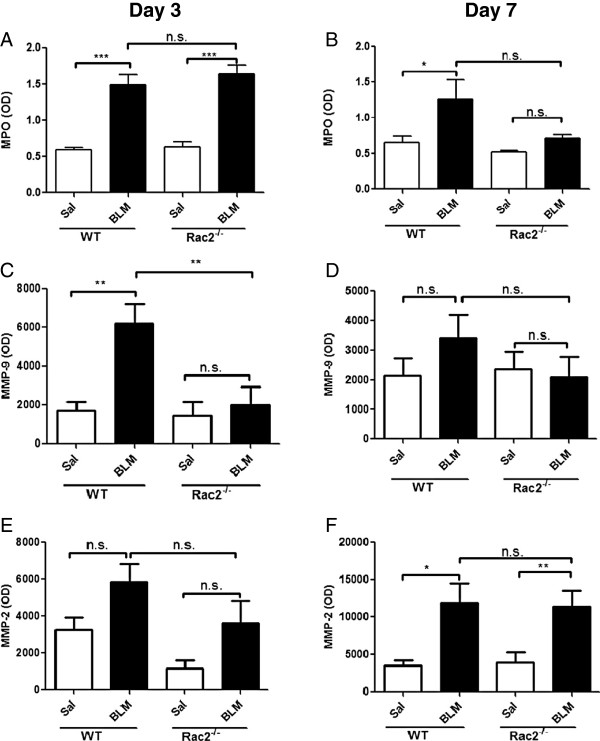
**MPO and MMP in BAL fluid*.*** MPO **(A and B)**, MMP-9 **(C and D)** and MMP-2 **(E and F)** levels in the BAL fluid on day 3 **(A, C, E)** and 7 **(B, D, F)** after intratracheal saline or bleomycin administration to WT and *rac2*^*-/-*^ mice. (n.s. not significant, *p < 0.05, **p < 0.01, ***p < 0.001, n = 5).

**Figure 3:**
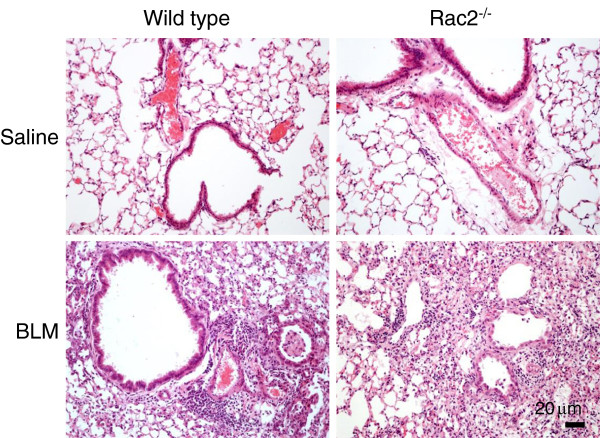
**Histological analysis of BLM-induced lung injury on day 7*.*** Representative images of H&E staining of lung sections 7 days after intratracheal saline or bleomycin administration to WT and *rac2*^*-/-*^ mice (in every condition 5 mice were analyzed by histology).

We next measured MPO activity in BAL samples. WT mice treated with BLM showed higher levels of MPO in BAL on day 3 and day 7 compared to saline-treated mice (Figure 2A and B n = 5). *Rac2*^*-/-*^ mice exhibited increased MPO activity on day 3 but not on day 7 (Figure 2A and B). There was no statistically significant difference in MPO between BLM-treated WT and *rac2*^*-/-*^ mice on day 3 or day 7. Histological analysis of the lungs of saline and BLM-treated WT and *rac2*^*-/-*^ mice on day 7 showed diffuse inflammatory cell infiltration in both groups of BLM-treated mice, but there were no clear differences between the two groups in the pattern or degree of inflammation (Figure 3).

We also examined the levels of matrix metalloproteinases in the BAL samples from these mice. BLM treatment increased MMP-9 levels in WT mice, but not in *rac2*^*-/-*^ mice, on day 3 (Figure 2C). MMP-9 levels returned to baseline by day 7 (Figure 2D). In contrast MMP-2 levels were increased in both WT and *rac2*^*-/-*^ mice on day 7, but not day 3, and there was no difference in MMP-2 levels between BLM-treated WT and *rac2*^*-/-*^ mice on day 3 (Figure 2E and F).

**Figure 4:**
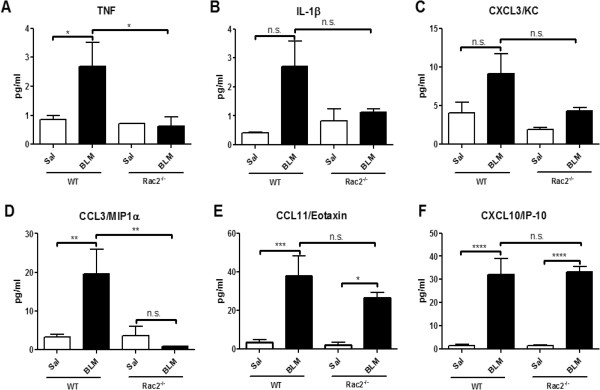
**Cytokine/chemokine levels in BAL fluid*.*** Levels of TNF **(A)**, IL-1β **(B)**, CXCL3/KC **(C)**, CCL3/MIP-1α **(D)**, CCL11/Eotaxin **(E)** and CXCL10/IP-10 **(F)**, were determined in BAL fluid 7 days after intratracheal saline or bleomycin administration to WT and *rac2*^*-/-*^ mice. (n.s. not significant, *p < 0.05, **p < 0.01, ***p < 0.001, ****p < 0.0001, n = 5).

In order to further understand the mechanisms for the defect in neutrophil migration to the airways in *rac2*^*-/-*^ mice treated with BLM, we evaluated the levels of various cytokines and chemokines in the BAL fluid. TNF was increased in WT mice following BLM administration, but not in *rac2*^*-/-*^ mice (Figure 4A, n = 5). IL-1β and CXCL3/KC showed a trend towards an increase in WT mice following BLM administration, but there were no statistically significant differences between any of the groups studies (Figure 4B and C, n = 5). CCL3/MIP-1α showed similar pattern with TNF (Figure 4D, n = 5). Finally CCL11/eotaxin and CXCL10/IP-10 were increased in both WT and *rac2*^*-/-*^ mice following BLM treatment and there was no difference between BLM-treated WT and *rac2*^*-/-*^ mice (Figure 4E and F, n = 5).

### Rac2 deficiency attenuates biological long-term consequences of BLM-induced lung injury

BLM-induced lung injury in mice is characterized by the development of extensive reversible fibrosis 15–20 days after a single treatment [22]. Fibrosis alters lung physiology and leads to significant morbidity and mortality in this model, before the process resolves. Since *rac2*^*-/-*^ mice showed altered early inflammatory response after lung injury, we next studied whether later events of BLM injury, including mortality and fibrosis, were also affected.

**Figure 5:**
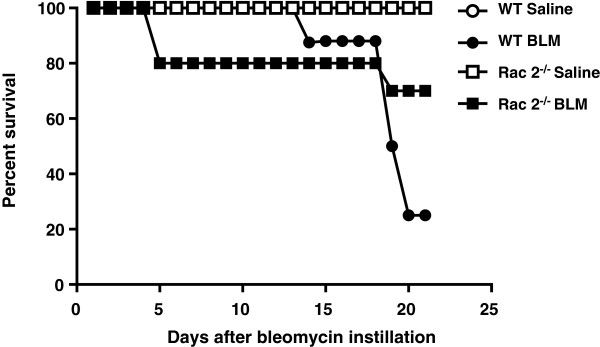
**Rac2 deficiency decreases mortality in bleomycin-induced pulmonary fibrosis*.*** Survival curves shown over 21 days for WT and *rac2*^*-/-*^ mice receiving BLM or saline (n = 13-22 per group).

To understand the function of Rac2 in the long-term effects of BLM-induced lung injury, WT and *rac2*^*-/-*^ mice were given BLM intratracheally and followed for 21 days. WT mice showed 70% mortality by day 21, while *rac2*^*-/-*^ mice showed significantly lower mortality (p < 0.005) (Figure 5). In order to further understand the functional role of Rac2 in BLM-induced lung injury, we analyzed lung physiology and markers of fibrosis in the mice that survived until day 21.

**Figure 6:**
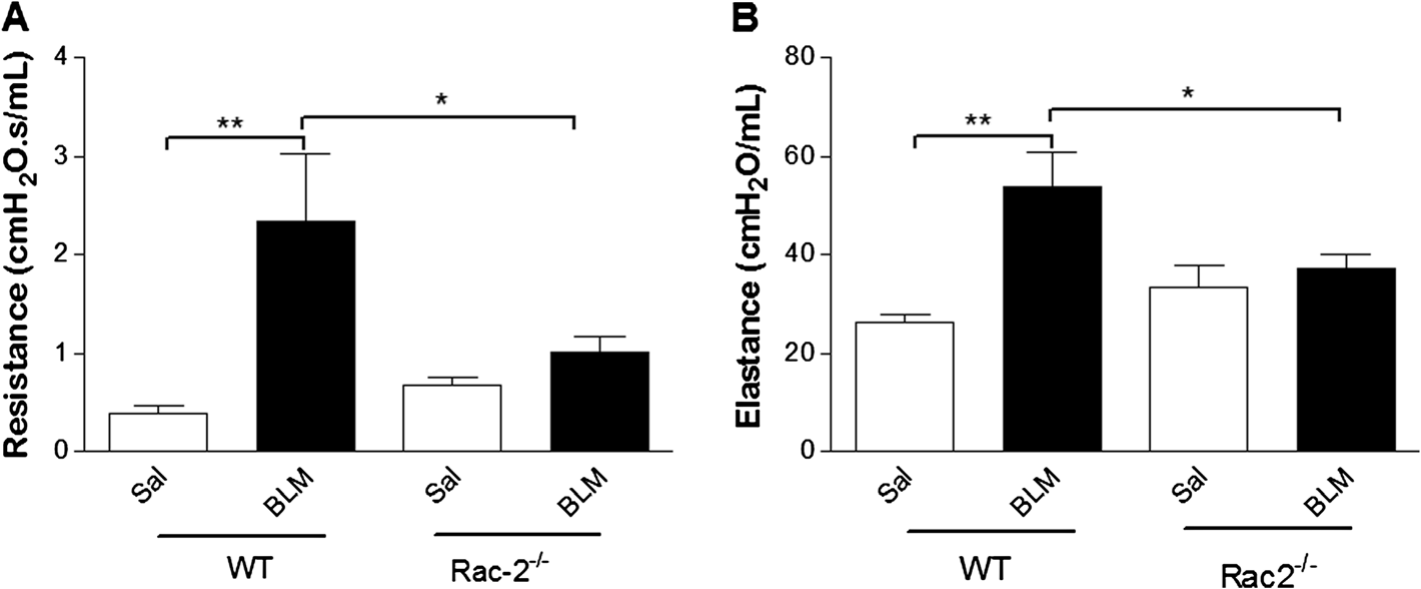
**Rac2 deficiency prevents BLM-included changes in pulmonary physiology*. *(A)** Resistance and **(B)** elastance 21 days after intratracheal saline or bleomycin administration to WT and *rac2*^*-/-*^ mice (*p < 0.05, **p < 0.01, n = 5-8).

WT mice that received BLM showed significantly increased airway resistance and elastance compared to saline-treated mice (p < 0.05) (Figure 6A and B, n = 8). In contrast, *rac2*^*-/-*^ mice that received BLM showed resistance and elastance levels that were similar to *rac2*^*-/-*^ mice receiving saline. There was also significant difference in resistance and elastance between BLM-treated WT and *rac2*^*-/-*^ mice.

The mortality and pulmonary physiology results indicate that BLM-induced injury is attenuated in *rac2*^*-/-*^ mice, and that in contrast to WT mice surviving to day 21, the injury still present in the *rac2*^*-/-*^ mice still surviving to day 21 does not affect lung physiology.

### Rac2 deficiency had no significant effect inflammation in BLM-treated mice surviving to day 21

We also performed histological analysis of lungs from surviving BLM-treated and saline-treated mice on day 21. Lungs were excised, fixed and then stained with H&E for morphology and inflammatory cell infiltration and PAS-D to identify mucin.

**Figure 7:**
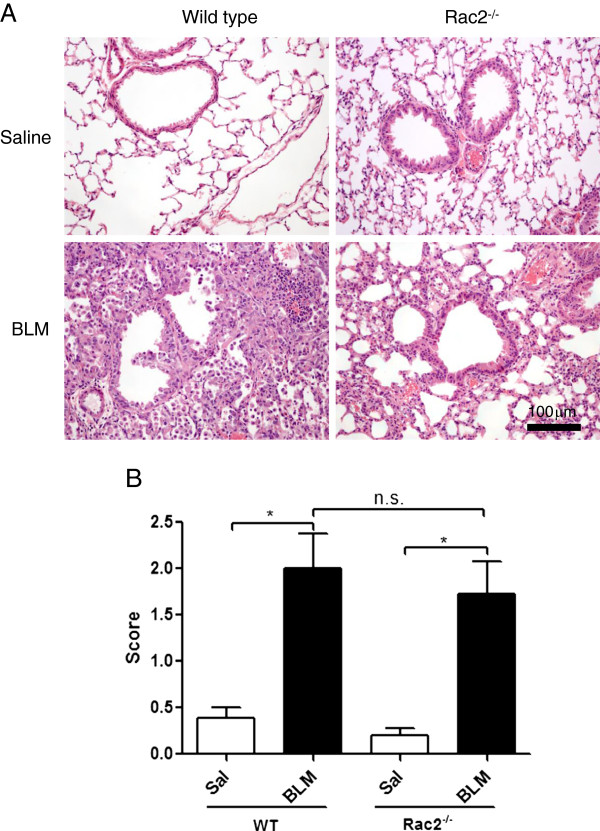
**Histological analysis of BLM-induced lung injury*. *(A)** Representative images from H&E staining of 21 days after intratracheal saline or bleomycin administration to WT and *rac2*^*-/-*^ mice **(B)** Inflammation score for saline and BLM-treated WT and *rac2*^*-/-*^ mice 21 days following saline or BLM administration (n.s. non-significant, *p < 0.05, n = 5-8).

Saline-treated WT and *rac2*^*-/-*^ mice (Figure 7A) showed normal architectural pattern of airways and alveoli, with an apparent increase in interstitial leukocytes evident in *rac2*^*-/-*^ mice. We hypothesize that the latter observation is the result of the modest peripheral neutrophilia that has been described in these animals [10], but we have no data to support this conclusion. Lung parenchyma of WT BLM-treated mice exhibited prominent perivascular, peribronchiolar and intra-alveolar inflammation, with increased numbers of alveolar macrophages (Figure 7A). The lungs of *rac2*^*-/-*^ BLM-treated mice showed a similar picture except that interstitial inflammation often had a more focal distribution. A semi-quantitative analysis (perivascular, peribronchial and intra-alveolar infiltrates were graded on a score of 0–3 for all the mice and these 3 numbers of every mouse were averaged and are presented as the inflammation score) showed no significant difference in inflammatory infiltrates between BLM-treated WT and *rac2*^*-/-*^ mice (Figure 7B).

**Figure 8:**
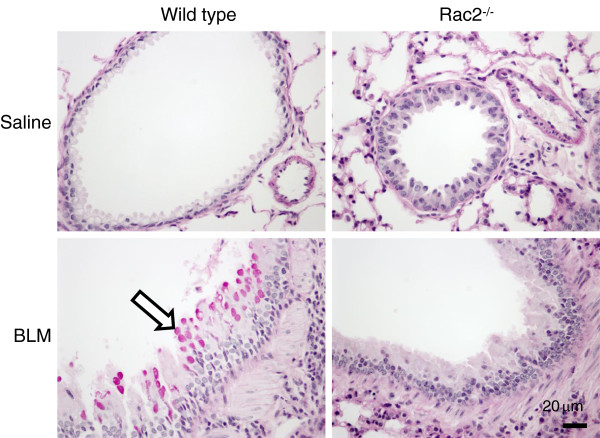
**Analysis of mucin expression following BLM-induced lung injury*.*** PAS-D staining of lung sections 21 days after intratracheal saline or bleomycin administration to WT and *rac2*^*-/-*^ mice (in each condition, 5–8 mice were analyzed by histology).

We also stained the lungs with PAS-D to identify mucin (Figure 8). WT BLM-treated mice displayed airway epithelium exhibiting increased numbers of mucin-containing epithelial cells consistent with goblet cell hyperplasia (arrow, fuchsia colored cells). In contrast, *rac2*^*-/-*^ BLM-treated mice showed airway epithelium with reduced numbers of mucin-containing epithelial cells compared to WT mice treated with BLM. In control saline-treated mice, there were essentially no mucin-containing epithelial cells in WT and *rac2*^*-/-*^ airway epithelia.

### Effect of Rac2 deficiency on fibrosis in BLM-treated mice surviving to day 21

To further assess the degree of fibrosis histologically, we analyzed lung sections from WT and *rac2*^*-/-*^ mice after Masson’s Trichrome staining to evaluate collagen deposition and measured total collagen in the lungs of these mice. We also performed immunohistochemical analysis with staining for αSMA and TGFβ.

**Figure 9:**
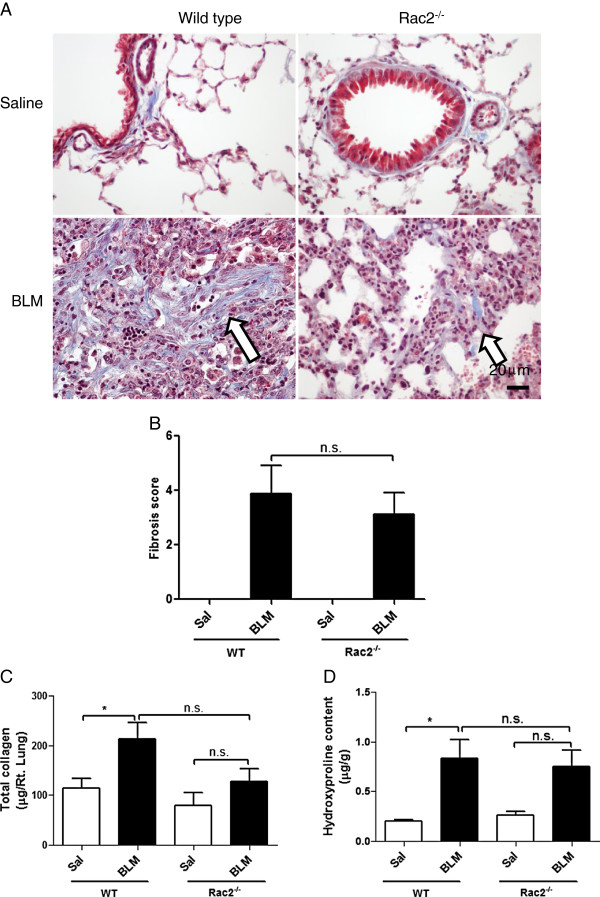
**Rac2 deficiency and BLM-induced fibrosis*. *(A)** Representative images of Masson’s trichrome staining of lung sections 21 days after intratracheal saline or bleomycin administration to WT and *rac2*^*-/-*^ mice (in every condition 5–8 mice were analyzed by histology). **(B)** Quantitation of lung fibrosis using Ashcroft’s criteria (5–8 mice were analyzed per group). Total collagen **(C)** and hydroxyproline content **(D)** of lungs 21 days were determined after intratracheal saline or bleomycin administration to WT and *rac2*^*-/-*^ mice (n.s. not significant, *p < 0.05, n = 5-8).

WT and *rac2*^*-/-*^ saline-instilled mice presented with normal lung parenchyma with an expected minimal level and distribution of collagen fibrils. In contrast, the lung parenchyma of WT and *rac2*^*-/-*^ BLM-treated mice showed increased collagen deposition compared saline-treated controls (arrows, Figure 9A lower panels). In general, BLM-treated *rac2*^*-/-*^ mice showed smaller patchy regions of collagen deposition compared to BLM-treated WT mice. However, evaluation of fibrosis by Ashcroft scoring as done before [15] showed no significant difference between BLM-treated WT and *rac2*^*-/-*^ mice (Figure 9B).

In order to determine fibrosis using biochemical approach, we estimated total lung collagen content using the Sircol Dye Reagent and hydroxyproline content using lung homogenates from saline and BLM-treated WT and *rac2*^*-/-*^ mice. We detected a significant increase in total lung collagen content and in hydroxyproline content in BLM-treated WT mice compared to saline-treated mice (p < 0.05, *n* =6), while *rac2*^*-/-*^ BLM-treated mice did not show significant increase in lung collagen or hydroxyproline content compared to saline-treated *rac2*^*-/-*^ animals (Figure 9C and D, n = 7-8 per group). However, BLM-treated *rac2*^*-/-*^ mice also showed no statistically significant difference in collagen or hydroxyproline content compared to BLM-treated WT mice.

**Figure 10:**
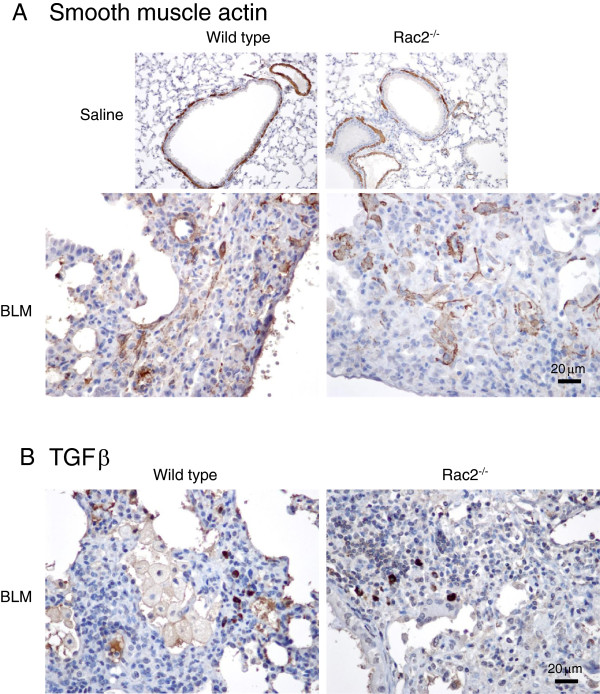
**αSMA and TGFβ immunohistochemistry*.*** Representative images showing immunostaining of αSMA, with arrows indicating the presence of myofibroblasts **(A)**, and TGFβ with arrows indicating TGFβ-positive leukocytes **(B)**, in lung sections 21 days after intratracheal saline or bleomycin administration to WT and *rac2*^*-/-*^ mice (in every condition 5–8 mice were analyzed by histology).

To further study the role of Rac2 in pulmonary fibrosis was also performed immunohistochemistry for αSMA and TGFβ. As shown in Figure 10, there were no differences in αSMA and TGFβ staining between BLM-treated WT and *rac2*^*-/-*^ mice on day 21.

## Discussion

Rac2 is a *ras*-related guanosine triphosphatase expressed mainly in hematopoietic cells, and is a crucial molecule regulating a diversity of neutrophil functions. We have previously shown that disruption of the gene encoding Rac2 significantly attenuated lung inflammation and injury in an immune complex-mediated acute lung injury model [13]. Injury in that model is mediated by Rac2 expression in hematopoietic cells. Although we did not identify the exact cell(s) mediating Rac2-dependent injury in that study, we speculated that neutrophils were primarily responsible. Accumulation of neutrophils in the BAL following immune complex-mediated acute lung injury was decreased, as it was also decreased in *rac2*^*-/-*^ mice in the BLM injury model we present here. We also showed here that there were differences in MMP-9 levels in the BAL between BLM-treated WT and *rac2*^*-/-*^ mice as well as differences in certain cytokine and chemokine levels. These inflammatory changes correlated with changes in lung physiology. There was however no significant difference in parenchymal inflammation seen by histology and in fibrotic markers between surviving BLM-treated WT and *rac2*^*-/-*^ mice on day 21 after BLM treatment. Our study also showed a large decrease in mortality of *rac2*^*-/-*^ mice compared to wild type mice following treatment with bleomycin. It is not clear whether the changes in inflammation or changes in lung physiology are responsible for this large decrease in mortality. Further work will be required to identify the mechanisms leading to Rac2-mediated mortality in the BLM model of lung injury. With the results presented here our group has shown that *rac2* gene deficiency prevents or attenuates tissue injury in both acute lung injury [13] and chronic lung injury (data presented here) models.

BLM-induced lung injury develops in two phases in mice. The first phase takes place over the first 9 days after BLM administration, and is characterized by inflammation and accumulation of inflammatory cells in the parenchyma and airways. The second fibrotic phase starts on day 9, with the presence of pro-fibrotic mediators, and leads to the development of overt fibrosis [22]. In our model, we have shown an increase in MMP-9 on day 3 along with an increase in the accumulation of neutrophils and macrophages in the airways on day 7 after BLM administration to WT mice. The MMP-9 and neutrophil increase, but not the increase in macrophage numbers, were absent from *rac2*^*-/-*^ mice treated with BLM. The exact reason for this is not clear at this time. Neutrophil chemotaxis [23] and the subsequent accumulation in the airways require Rac2 function, and the lack of neutrophil accumulation may be an early event leading to these differences in *rac2*^*-/-*^ mice.

Neutrophil accumulation and mediator release have been implicated in the pathophysiology of BLM-induced lung injury. Lack of CXCR2, the receptor for CXCL3/KC, one of the main neutrophil chemotactic agents in mice, resulted in decreased lung fibrosis along with decreased accumulation of neutrophils in the airways [24]. That study showed that CXCL3/KC levels increased 6 h after BLM treatment and returned to normal by day 8. This may explain why we did not see significant changes in CXCL3/KC levels in our mice when we analyzed them on day 7. It is interesting to note that in the same study the numbers of neutrophils in the parenchyma, as assessed by the levels of MPO in the lung, were not decreased, although tissue injury was decreased. In our study also we did not see a significant difference in inflammatory cell infiltration in the lung tissue on day 7 by histology between WT and *rac2*^*-/-*^ mice, although there was significant difference in the numbers of neutrophils in the BAL on day 7. Transmigration of neutrophils into the airways might facilitate fibrosis through the release of mediators that can activate TGFβ. For example it has been shown that lack of elastase prevented TGFβ activation in the airways and decreased the development of fibrosis following bleomycin administration [20]. In our study we did not see significant differences in TGFβ positive cells on day 21, but we did not analyze TGFβ expression at earlier time points to know if there were any differences.

We showed significant differences in TNF and CCL3/MIP-1α between BLM-treated WT and *rac2*^*-/-*^ mice. Both of these mediators have been implicated in the pathophysiology of bleomycin-induced injury. The degree of bleomycin-induced injury correlates with TNF upregulation in the lungs of different strains of mice [25] and the presence of soluble TNF has been shown to be required for the development of fibrotic lesions in the lungs following bleomycin administration [26]. In addition TNF has been shown to be mediating the increased expression of CCL3/MIP-1α in the lungs of BLM-treated mice [27]. CCL3/MIP-1α has been shown to be elevated throughout the time course of bleomycin-induced lung injury and is required for injury to develop [28]. CCL3/MIP-1α recruits macrophages to the lung through the interaction with CCR5 and may increase the expression of TGFβ in the airways. Lack of these two inflammatory mediators in *rac2*^*-/-*^ mice may be responsible for the physiological and other changes described in this manuscript.

A number of other mediators may also be involved in the recruitment of neutrophils and the neutrophil-mediated effects in the BLM model. IL-1β is increased in the BAL of humans with idiopathic lung fibrosis [29]. IL-1β was also induced by bleomycin in mouse lungs [30] and lung injury was attenuated when IL-1β activity was decreased [30]. In our study we only saw a trend towards decreased levels of IL-1β in *rac2*^*-/-*^ mice compared to WT mice following BLM treatment. Chemokines that activate CXCR3, such as CXCL10/IP-10 and CXCL9/MIG, have a protective role and can limit fibrosis in the lungs [31,32]. CCL11/eotaxin has also been shown to mediate another pathway that leads to BLM-induced lung fibrosis [33]. Our data indicate that these pathways may not be affected in the absence of Rac2. There are finally also other cytokines that have been shown to mediate BLM-induced lung injury. For example, IL-17A is required for BLM-induced fibrosis [29] and its main effect in the model may be recruitment of neutrophils. Neutrophils in the airways of mice with BLM injury produce IL-18, and inhibition of IL-18 can prevent fibrosis [30]. IL-18 was also increased in the lungs of patients with lung injury after receiving BLM. It would be interesting to also study changes in these inflammatory mediators in BLM-treated Rac2 mice to better understand the mechanism of protection.

Other members of the Rho family of GTPases have also been implicated in lung fibrosis. A RhoA inhibitor decreased accumulation of macrophages and neutrophils in the airways of BLM-treated mice and also BLM-induced fibrosis [34]. In this case the effect is more likely through inhibition of the proliferative effects of RhoA in lung fibroblasts [35]. In addition Rac1 expression in cutaneous fibroblasts in required for the development of BLM-induced skin fibrosis [36]. This is an important family of signaling molecules and there has been significant effort in identifying inhibitors for these GTPases. This fact increases the translational potential of these studies, since these inhibitors may be proven to be valuable as therapeutic options for lung fibrotic diseases.

The levels of MMP-2 and MMP-9 in the BAL fluid of WT mice given BLM correlate with the levels in lung tissues, and reportedly peak at day 4 [37]. In our case we observed that the levels of MMP-9 were decreased compared to WT responses on day 3 in *rac2*^*-/-*^ mice, suggesting that MMP-9 production was lacking in the knockout mice. In our earlier study on acute lung injury, we determined that the main cell type responsible for generating MMP-2 and MMP-9 in lung tissues was the tissue macrophage [13]. These findings indicate that macrophages may be responsible for elevated production of MMP-2 and MMP-9 in early stages of lung injury. Further studies are needed to determine if reduced Rac2 activity in macrophages leads to reduced secretion of MMPs, or if reduced recruitment of neutrophils to the airways of BLM-treated *rac2*^*-/-*^ mice results in decreased macrophage activation.

Macrophages have also been implicated in the pathophysiology of BLM-induced injury in mice [21,38]. In our study we did not see a difference in macrophage accumulation in the airways in the early stages of BLM-induced injury in *rac2*^*-/-*^ mice. However, Rac2 is important for macrophage function, and it is possible that decreased activity and or altered activation status of the recruited macrophages may have a role in the effects we describe. A role for Rac1 in the inflammatory, but not fibrotic, phase of asbestos-induced lung injury in mice has been shown in a mouse strain with conditional, myeloid-specific gene deletion of Rac1 [39]. This observation suggests that granulocyte/macrophage-expressed Rac1 regulates inflammatory processes, but has little impact on fibrotic lesions arising from asbestos exposure. Although a different model was used in our study, our findings show an additional role for Rac2 in lung physiology and mortality associated with fibrosis, which has not been reported before.

## Conclusions

This manuscript shows that *rac2* deficiency decreases BLM-induced mortality from 70% in wild type mice to less than 30% in Rac2 knockout mice. Rac2 activation also plays an important role in BLM-induced alterations in respiratory physiology and may also mediate the development of early airway inflammation in response to inhaled BLM. We conclude that Rac2 mediates many physiological changes seen in the lungs that may be related to BLM-induced mortality.

## Abbreviations

αSMA: α-smooth muscle actin; BAL: Bronchoalveolar lavage; BLM: Bleomycin; H&E: Hematoxylin and eosin; MMP: Matrix metalloproteinases; PAS-D: Periodic-acid Schiff diastase; Rac2: *ras*-related C3 botulinum toxin substrate 2; WT: Wild type.

## Competing interests

The authors declare that they have no competing interests.

## Authors’ contributions

NA performed the experiments describing mortality and fibrosis in the BLM model and generated the first draft of the manuscript, LP and KC performed the histological analysis, BT advised on the use of animal models, DC and CD performed the experiments for analysis of MMP, PL and HV supervised the study and wrote the manuscript with input from the other authors. All authors read and approved the final manuscript.
